# 265. Rocky Mountain Spotted Fever Encephalopathy

**DOI:** 10.1093/ofid/ofab466.467

**Published:** 2021-12-04

**Authors:** Sangeetha Isaac, Mohammed Afraz Pasha, Jean H Vincent, Khushdeep Chahal

**Affiliations:** 1 North Alabama Medical Center, Florence, Alabama; 2 North Alabama Internal Medicine Residency Program, Florence, Alabama

## Abstract

**Background:**

Rocky mountain spotted fever (RMSF) is a rickettsial disease with incidence of 11 per million and is rarely associated with encephalopathy. Here we discuss a patient with RMSF encephalopathy, highlighting the natural course.

**Methods:**

A 54 year old man with history of hypertension and chronic progressive external ophthalmoplegia, presented with waxing and waning confusion, headache, slurred speech, agitation and difficulty swallowing. He was afebrile and hemodynamically stable. Investigations showed leukocytosis of 15400 and mild transaminitis. Computed-tomography (CT) head was unremarkable. Lumbar puncture revealed normal pressure. Cerebrosopinal fluid (CSF) analysis was notable for WBC 7, glucose 76 and moderately elevated total protein 114. Urine drug screen was negative. Blood, fungal, and CSF cultures were sent and empiric therapy with vancomycin, ceftriaxone, ampicillin and acyclovir commenced, for suspected encephalitis. High dose solumedrol 1gm/day was given due to suspicion of autoimmune encephalitis. MRI brain showed cerebral atrophy. There was slight abnormal FLAIR/T2 signal within the medial aspect of the temporal lobes, right more than left.

**Results:**

Occupational history revealed that he was a logger by profession, which steered our focus on tick borne diseases. Extensive serologic evaluation was requested and RMSF IgG titres came back positive at 1:512. Doxycycline was added, while ampicillin and ceftriaxone were discontinued. With doxycycline, patient made remarkable recovery and was discharged home well. However, he returned within 48 hours with recurring encephalopathy. His clinical presentation remained convincing for RMSF encephalitis, with the natural course of the illness spanning over weeks, with waxing and waning symptoms. Patient was managed with IV doxycycline for 72 hours following which he returned to his baseline mental status.

Figure 1. MRI findings

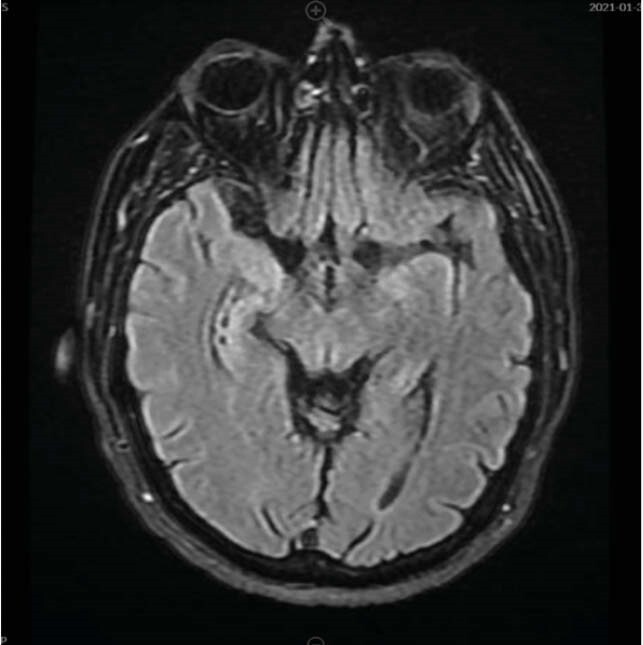

Figure 2. Serological investigations

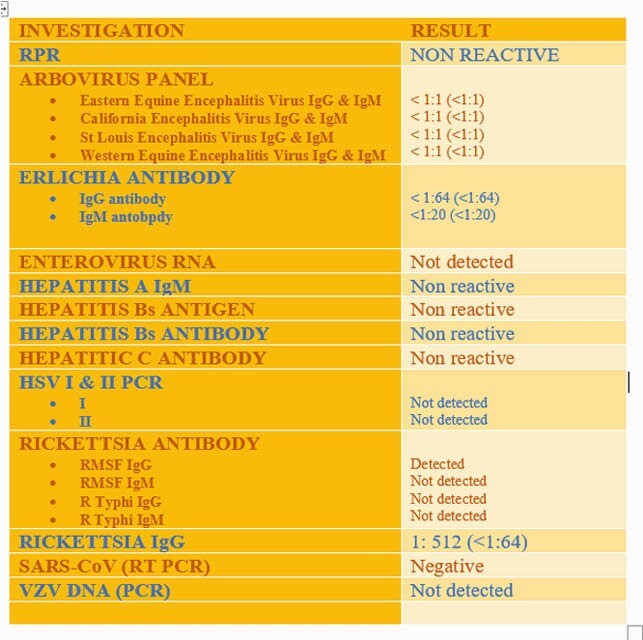

Figure 3. CSF studies

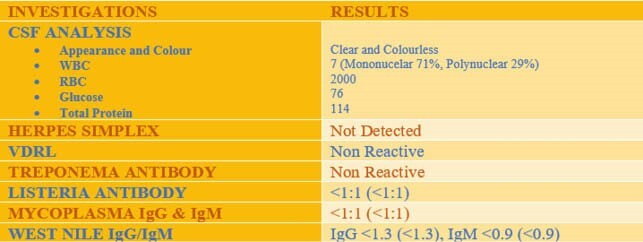

**Conclusion:**

Patient’s occupation played a pivotal role in establishing diagnosis. In RMSF, IgM and IgG antibodies appear 7 to 10 days after the onset of the illness, and a fourfold rise in IgG is diagnostic of seroconversion and recent illness. Patient’s waxing and waning symptoms, persisting for weeks and remarkable response to doxycycline, are typical features of RMSF encephalitis.

**Disclosures:**

**All Authors**: No reported disclosures

